# Neurological update: gliomas and other primary brain tumours in adults

**DOI:** 10.1007/s00415-017-8652-3

**Published:** 2017-11-02

**Authors:** Sebastian Brandner, Zane Jaunmuktane

**Affiliations:** 1 Division of Neuropathology, National Hospital for Neurology and Neurosurgery, University College London NHS Foundation Trust, Queen Square, London, WC1N 3BG UK; 2 Department of Neurodegeneration, Institute of Neurology, University College London, Queen Square, London, WC1N 3BG UK; 3 Department of Molecular Neuroscience, Institute of Neurology, University College London, Queen Square, London, WC1N 3BG UK

**Keywords:** IDH, ATRX, TERT, EGFR, BRAF, Histone, Mutation, Astrocytoma, Oligodendroglioma, Glioblastoma, Ependymoma, BRAF inhibitor, Tumour heterogeneity, MGMT, Temozolomide

## Abstract

The emerging understanding of molecular changes in a wide range of brain tumours has led to a significant shift in how these tumours are diagnosed, managed and treated. This article will provide a hands-on overview of the relevant biomarkers and their association with newly defined biological tumour entities.

## Introduction

The traditional approach to diagnose brain tumours is the examination of a histological specimen. Conventionally, pathologists make the histological diagnosis by assessing morphological features of cellular atypia, variation of nuclear size (anisonucleosis), shape (pleomorphism), mitotic activity, cell density, characteristic architectural patterns, vascular properties, and cell necrosis. With the exception of histone-mutant diffuse midline gliomas, these cytoarchitectural characteristics are taken into account when assigning a malignancy grade to the tumour according to the WHO classification scheme [28]. This is still the mainstay of the diagnostic approach and is suitable and adequate for the vast majority of tumours, including brain tumours. While a small proportion of brain tumours in adults is caused by germ line mutations and is associated with a number of syndromes, the majority of adult gliomas develop sporadically. Recent combined efforts by large research consortia have, however, led to the discovery of a number of key mutations, chromosome copy number variations and epigenetic alterations in a range of intrinsic brain tumours [27], challenging the clinical relevance of the traditional diagnostic approach. The first most detailed molecular subclassification of brain tumours has been achieved with medulloblastomas, a malignant, predominantly paediatric, brain tumour arising in the cerebellum, whereby the gene expression profile reflects the activation of distinct signalling pathways and correlates much better with clinical outcome and therapy response than the conventional subclassification approaches based on histological features [54]. In adults, the most common intrinsic brain tumours are gliomas, such as glioblastomas, astrocytomas, oligodendrogliomas and ependymomas, arising throughout the neuraxis and displaying variable biological behaviour. The discovery of mutations in specific genes has revolutionised our understanding of the pathogenesis of many type of glioma and has subsequently led to a biomarker-driven classification which in current practice not only supplements, but increasingly overrides the histological diagnosis. Another important development is the recognition of clinically relevant, molecularly defined tumour classes. Mounting evidence indicates that for certain nosological entities, for example, IDH-mutant astrocytomas [40] or ependymomas [31], the molecular profile much better reflects the biological behaviour, superseding the relevance of conventional histological grading, which is based on histological features.

The focus of this review article is limited to primary, intrinsic brain tumours occurring in adults. Each molecularly defined diagnostic group, i.e. IDH-mutant astrocytomas or oligodendrogliomas, histone-mutant gliomas, BRAF-mutant gliomas and ependymomas will be discussed separately with an emphasis on the specific molecular alterations in each group and their clinical relevance.

## IDH-mutant gliomas: astrocytomas, glioblastomas and oligodendrogliomas

### Genetics and pathology

**Fig. 1:**
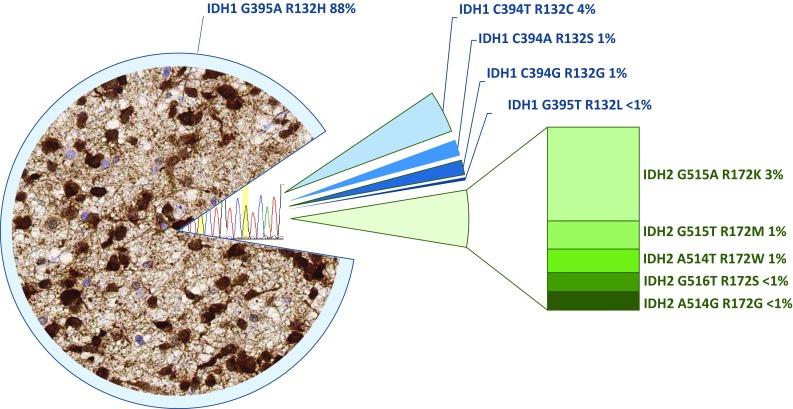
Frequency of *IDH1* and *IDH2* mutations in a cohort of 747 oligodendroglial and astrocytic tumours (extracted from the data in [19]) and two added rare *IDH2* mutations. The left part of the graph shows a typical histological image of immunostaining in an *IDH1* R132H mutant astrocytoma with an antibody detecting this specific mutation. Approximately 90% of all IDH-mutant tumours and 95% of *IDH1* mutations are detected with this antibody. The remaining *IDH1* mutations and all *IDH2* mutations are most commonly detected by sequencing the hotspot on codon 132 (*IDH1*) and 172 (*IDH2*) (right part of the figure). The frequency of IDH-mutant gliomas rapidly decreases with the age of the patient. The probability of an alternative *IDH* mutation is < 6% in a 50-year-old patient and decreases to < 1% in patients aged > 54 years [7]

**Fig. 2:**
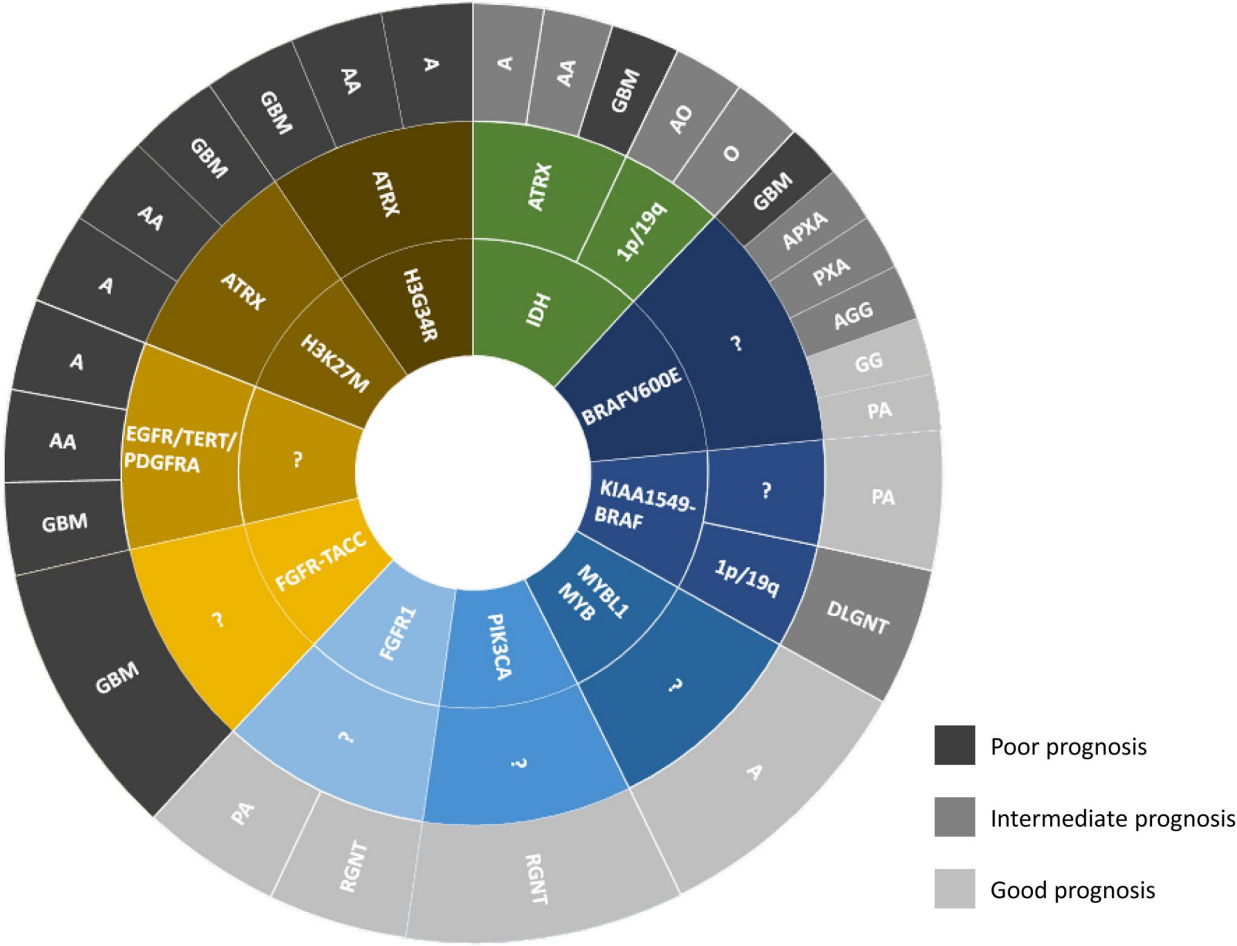
Simplified scheme of known genetic alterations in the most common glial and glioneuronal tumours. The inner circle shows the presumed driver mutation, such as *IDH* (green), histone H3.3 K27M, G34R, *FGFR–TACC* fusion (brown), or *BRAF alterations* (dark blue), *MYB/MYBL1*, *PIK3CA* and *FGFR1* (lighter blue shades). The middle circle shows known additional mutations that are associated with the respective tumour entities. For example, in the group of IDH-mutant tumours, the 1p/19q codeletion defines the oligodendroglioma, whilst the ATRX mutation defines the IDH-mutant astrocytoma. The IDH-wildtype glioblastoma (light brown) currently does not have a defined driver mutation, but it contains a combination of signature alterations such as *EGFR* or *PDRGRA* amplifications, *TERT* promoter mutation and others. The outer circle shows the histological diagnosis with the abbreviations corresponding to the following histological entities: *A* astrocytoma, *AA* anaplastic astrocytoma, *GBM* glioblastoma, *O* oligodendroglioma, *AO* anaplastic oligodendroglioma, *PXA* pleomorphic xanthoastrocytoma, *APXA* anaplastic pleomorphic xanthoastrocytoma, *GG* ganglioglioma, *AGG* anaplastic ganglioglioma, *PA* pilocytic astrocytoma, *DLGNT* diffuse leptomeningeal glioneuronal tumour, *RGNT* rosette forming glioneuronal tumour. The grey shades for each histological diagnosis indicate the prognosis: light grey corresponds to good prognosis and darkest grey corresponds to poorest prognosis. Note that tumours with identical histological diagnoses, depending on their underlying genetic alteration, can show different biological behaviour and thus have a different prognosis. For example, H3 K27M mutant gliomas (brown) can have a low- or high-grade histological appearance but all have a poor prognosis. Conversely, BRAF-mutant gliomas cover a wide spectrum prognoses ranging from benign to highly malignant tumours. The diagram is for illustrative purposes and does not reflect frequencies of any of these entities

The discovery of mutations in the isocitrate dehydrogenase genes 1 and 2 (*IDH1*, *IDH2*) in 2008 was a major breakthrough towards molecular biomarker-driven diagnosis of adult gliomas [34]. Mutations on codon 132 or 172 of the *IDH1* and *IDH2* genes, respectively, results in “neo-enzymatic activity” with the production of the novel oncometabolite 2-hydroxyglutarate [11] causing widespread methylation of the tumour cell DNA [55] and altered regulation of histone methylation [9]. IDH-mutant gliomas mainly arise in young adults in their second to fourth decade of life and are rare in people over 55 [7]. *IDH* mutations occur in two classes of gliomas, astrocytomas and oligodendrogliomas. IDH-mutant astrocytomas over time progress to IDH-mutant anaplastic astrocytomas and IDH-mutant glioblastomas, previously also known as secondary glioblastomas. However, the IDH-mutant anaplastic astrocytoma and IDH-mutant glioblastoma can also develop de novo with no previous clinical and radiological evidence of a lower grade glioma. The majority (90%) of both astrocytomas and oligodendrogliomas carry a specific point mutation (R132H) in the *IDH1* gene [19], which can be detected immunohistochemically with a mutation-specific antibody [5]. The detection of the other mutations (Fig. 1) requires sequencing of the *IDH1* and *IDH2* genes [20, 36]. The presence of an *IDH1* or *IDH2* mutation is also required for the diagnosis of oligodendroglioma and anaplastic oligodendroglioma. The previously known entity of oligoastrocytoma was defined on histological grounds only and is now extinct [44], as there is robust evidence that the *IDH* mutations segregate either with the chromosomal codeletion of 1p/19q in oligodendrogliomas, or with a loss of function mutation in the *ATRX* gene (alpha thalassaemia/mental retardation syndrome X-linked) in astrocytomas [41] (Fig. 2). Combined 1p/19q loss (also known as 1p/19q codeletion) can be tested with a number of molecular methods, such as fluorescent in situ hybridisation (FISH), qPCR and various array technologies. Mutations in the *ATRX* gene are routinely tested in most laboratories by immunostaining for the ATRX protein, which detects loss of expression resulting from the majority of *ATRX* gene mutations [23, 25]. However, a small proportion of mutations in the *ATRX* gene does not result in the loss of protein expression, and thus are not detectable by immunohistochemistry. Sequencing of the *ATRX* gene would be desirable, but is currently not practical in routine diagnostics in most laboratories, due to the large size of the *ATRX* gene and the wide range of mutation sites. Diagnostically useful is the additional testing for mutations in the promoter region of telomerase reverse transcriptase (*TERT*), which leads to an upregulation of the telomerase complex activity, increasing tumour cell survival. Two hotspots in the *TERT* promoter (C228T or C250T) are strongly associated with oligodendrogliomas. Whilst *TERT* promoter mutations are also seen in a proportion of IDH-wildtype glioblastomas and other tumours, they are generally not seen in IDH-mutant astrocytomas [24]. IDH-mutant glioblastomas almost never show EGFR amplification, unlike their IDH-wildtype counterparts (see below) [41]. Of note, *IDH* mutations also are mutually exclusive with mutations in the *BRAF* gene (see below and Fig. 2).

### Clinical relevance

The prognosis is currently determined more by the patient’s age, performance status and the molecular genetic profile of IDH-mutant tumours, than by standard treatment options such as surgery, radiotherapy and chemotherapy [57]. The combination of *IDH* mutation and 1p/19q codeletion predicts a favourable response to upfront combined radiochemotherapy. The importance of a 1p/19q codeletion, in particular, in anaplastic oligodendrogliomas has been demonstrated in several prospective phase 3 trials [56]. While IDH-mutant astrocytomas have a poorer prognosis than IDH-mutant, 1p/19q codeleted oligodendrogliomas, IDH-mutant glioblastomas have a considerably better prognosis than IDH-wildtype glioblastomas [59]. Importantly, there appears to be little difference in overall survival between IDH-mutant WHO grade II astrocytomas and WHO grade III IDH-mutant anaplastic astrocytomas, in this instance challenging the current histological approach in assigning a malignancy grade to tumours [40]. A targeted therapy in the form of a “vaccine” has been developed against IDH-mutant tumour cells and is currently being rolled out in a clinical trial [48]. The production of a specific oncometabolite by IDH-mutant tumours has also led to studies measuring 2-hydroxyglutarate in body fluids, such as plasma and urine [14], and by imaging techniques [8] to assess disease progression and/or response to therapy.

## IDH-wildtype gliomas: glioblastomas and their precursors

### Genetics and pathology

Glioblastoma is the most common primary malignant brain tumour in adults. Glioblastoma shows a variable, but usually characteristic histopathological appearance and poses no diagnostic challenge in most cases. The morphological diagnostic features include tumour necrosis and/or microvascular proliferation (vessels with multilayered abnormal endothelium) in a diffusely infiltrating, mitotically active astrocytic tumour. A challenge to diagnose glioblastoma histologically arises from small biopsies, which may contain only the infiltration zone of the tumour. It is increasingly important in clinical practice to recognise diffusely infiltrating gliomas, which do not show high-grade features by imaging or histology, yet represent early forms of IDH-wildtype glioblastoma [39] (Fig. 2). Prior to the discovery of *IDH* mutations as biomarkers of diffuse astrocytomas, such “early” glioblastomas were morphologically indistinguishable from other forms of astrocytomas but showed a rapid progression, posing a significant challenge to the WHO classification system. Currently, no specific mutation has been identified in IDH-wildtype glioblastomas that could serve as a useful biomarker, in the same way that *IDH* mutations do for oligodendrogliomas and astrocytomas. Instead, an increasing number of genetic and epigenetic alterations are discovered in IDH-wildtype glioblastomas, indicating that the nosological entity of an IDH-wildtype glioblastoma encompasses tumours with multiple distinct molecular signatures. The molecular alterations in IDH-wildtype glioblastomas include mutations in the *TERT* promoter [24], chromosome 10q loss, 7p gain or *EGFR* amplification, (some with an additional *EGFR vIII* mutation), *ID2*, *MYCN* and *PDGFRA* amplifications and *CDKN2A/B* deletions [45]. Molecular testing for these alterations can be helpful in identifying glioblastomas even in small samples, which do not demonstrate histological diagnostic criteria for glioblastoma (Fig. 2). It is now well established that the *p53* gene, one of the first genes found to be frequently altered in gliomas, can be mutant in both IDH-wildtype and IDH-mutant glioblastomas [25], and therefore the detection of *p53* mutations is diagnostically and prognostically not relevant [2]. A small proportion of IDH-wildtype glioblastomas harbour a *BRAF* V600E point mutation (see below) or *FGFR–TACC* fusions [13].

### Clinical relevance

The molecular alterations mentioned above can under certain circumstances be diagnostically useful, and thus may be important for adequate treatment planning. However, none of these tests have any proven prognostic or predictive significance on their own. The only known biomarker indicating a benefit from treatment with a specific alkylating agent (temozolomide) is the methylation status of the MGMT promoter. MGMT is a DNA repair protein which repairs chemotherapy-induced alkylation at the O6 position of guanine, thus counteracting the effects of alkylating chemotherapy. Methylation of the MGMT promoter is thought to silence gene expression and therefore reduce the repair activity of the protein. MGMT promoter methylation is associated with prolonged progression free and overall survival in patients with glioblastoma, who are treated with temozolomide [51, 56]. It must be noted that MGMT promoter methylation can occur in many other cancers and therefore cannot be used as a diagnostic marker. There are a number of ongoing clinical trials and emerging trials for both newly diagnosed and recurrent glioblastomas, using inhibitory drugs targeting specific cellular pathways or immunotherapy-based approaches with monoclonal antibodies or dendritic cell-derived vaccines. However, the existence of several molecularly and prognostically distinct subtypes of IDH-wildtype glioblastomas, and their highly infiltrative nature may contribute to the difficulties in finding an effective and tailored treatment for them.

## BRAF-mutant gliomas

### Genetics and pathology

Somatic mutations in the *BRAF* gene were initially discovered in melanomas and in a wide range of other cancers including colorectal and ovarian tumours as early as 2002 [12]. A decade later, mutations in the *BRAF* gene, the V600E point mutation in particular, have been demonstrated in a range of low-grade IDH-wildtype glial and glioneuronal tumours [47], and also are increasingly recognised in malignant variants [47, 53]. Mutations in the *BRAF* gene activate the MAP kinase pathway cascade, stimulating cell growth. Tumours in which this point mutation is most commonly found are pleomorphic xanthoastrocytoma (PXA, 60%), ganglioglioma and gangliocytoma (30%), subependymal giant cell astrocytoma (SEGA, 40%), desmoplastic infantile glioma (10%) and pilocytic astrocytoma (5% infratentorial and 20% supratentorial) [3, 10]. Unlike the well-defined molecular classes of IDH-mutant tumours, the V600E point mutation in the *BRAF* gene occurs only in a subset of these nosological tumour entities. In other words, while the presence of *IDH* mutation is required for the molecular diagnosis of an astrocytoma or oligodendroglioma, in tumours such as ganglioglioma, pleomorphic xanthoastrocytoma or pilocytic astrocytoma the *BRAF* V600E mutation is not always present. The *BRAF* V600E mutation has also been documented in a morphological variant of glioblastoma (epithelioid glioblastoma). It is being debated if epithelioid glioblastoma represents the most malignant form of PXA [53].

Currently, the detection of the *BRAF* V600E mutation is diagnostically useful for confirmation of a neoplastic process, but it does not identify a specific nosological entity. With personalised, targeted therapy in mind, in the future it may be more relevant to test for this mutation rather than providing a detailed histological characterisation of these brain tumours.

Apart from a single substitution V600E mutation, other mutations in the *BRAF* gene, which can occur in IDH-wildtype gliomas and glioneuronal tumours, include rearrangements, duplications and fusions with other genes and their detection may be diagnostically helpful. *KIAA1549*-*BRAF* fusions are particularly frequently seen in pilocytic astrocytomas, a low grade, predominantly paediatric tumour arising most commonly in the posterior fossa. This fusion mutation is most common (approximately 85%) in posterior fossa pilocytic astrocytomas, and less frequently (50–60%) in hemispheric and diencephalic locations [10]. Those pilocytic astrocytomas which do not harbour *KIAA1549*–*BRAF* fusion mutations, have been found to have mutations in genes encoding constituents of the MAP kinase pathway, including *FGFR1* and *NTRK* gene family*, NF1, PTPN11, KRAS* and *RAF1* [10, 21, 60].

### Clinical relevance

Inhibition of the activating effects caused by the *BRAF* V600E mutation was the rationale for developing inhibitor drugs interrupting the BRAF/MEK component of the MAP kinase pathway. Target-specific drugs (e.g. Vemurafenib, Dabrafenib) were developed and initially approved for the treatment of *BRAF* V600E mutant melanomas, and have since been trialled first in malignant, and more recently in low-grade *BRAF* V600E mutant brain tumours [1, 35]. The development of drug resistance has led to the generation of MEK inhibiting drugs (e.g. Trametinib, Cobimetinib) which seem to be effective in combination with BRAF inhibitors [16, 18]. BRAF fusions have no known prognostic value, however, tumours bearing these mutations may in the future benefit from MAP kinase pathway inhibitors [43].

## Histone-mutant gliomas

### Genetics and pathology

Mutations in the histone genes have recently been demonstrated in several malignant tumours, including high-grade gliomas. Several histone gene families exist, and the most commonly affected histone genes in brain tumours encode histone variant H3.3 (*H3F3A, H3F3B*), and less commonly histone variant H3.1 (*HIST1H3B*, *HIST1H3C*) [22]. The identified missense mutations affect three specific amino acids in the N-terminal tail of histone H3 (i.e. K27M, G34R and G34V). These mutations are highly specific, and are considered as “driver” mutations, i.e. tumour-initiating [49]. The histone H3.3 K27M mutation almost exclusively occurs in CNS tumours of the midline (thalamus, basal ganglia, brain stem and spinal cord) mostly in children, but as the mutation can be easily detected by immunohistochemistry with a histone H3.3 K27M mutation-specific antibody, it is increasingly frequently identified also in adults [6]. The H3 K27M mutant diffuse midline gliomas of the brain stem are also known as diffuse intrinsic pontine gliomas (DIPG). H3 K27M mutant gliomas correspond to WHO grade IV (i.e. the most malignant grade) irrespective of their histological appearance. Therefore, these tumours form the first entity in the WHO classification in which the molecular signature, rather than the morphology, defines the grade of malignancy. Instead tumours outside the midline, i.e. in the cerebral hemispheric regions, more commonly carry the histone H3.3 G34R or rarely G34V mutations. The majority of these mutations are found in the *H3F3A* gene. A proportion of histone-mutant tumours also harbour mutations in the *ATRX* gene. These often result in the loss of nuclear protein expression in tumour cells, which can be detected by immunohistochemistry and thus facilitate a more rapid histological diagnosis (Fig. 2).

### Clinical relevance

For paediatric patients with diffuse intrinsic pontine gliomas (DIPG), the largest group of histone-mutant tumours, treatment options are very limited with no effective conventional chemotherapeutic agents, and radiation therapy being the standard of care, with a poor survival rate of less than 10% 2 years after diagnosis [26]. Although as of now, there is no specific treatment available for histone-mutant gliomas, the discovery of mutations in histone genes and ongoing further research into the underlying mechanisms has laid the foundation for the development of targeted therapies with the aim to inhibit histone methylase and demethylase (reviewed in [26]). H3.3 K27M mutations lead to a global reduction of trimethylated H3K27 (H3K27me3). The level of trimethylation at this residue is regulated by the methylating enzyme EZH2 and the de-methylating JMJD3. High EZH2 levels correlate with poor overall survival, suggesting it as a potential target, and experimental preclinical studies are promising [29]. Another strategy focuses on the inhibition of the demethylase JMJD3; such a treatment has been successful in preclinical studies (reviewed in [50]).

## Other low-grade glial and glioneuronal tumours

### Genetics and pathology

This paragraph summarises a number of rare, histologically diverse tumours, comprising dysembryoplastic neuroepithelial tumour (DNET), the above-mentioned ganglioglioma with its histological and grading variants (gangliocytoma, a predominantly neuronal variant, and anaplastic ganglioglioma, a malignant variant). Other rare entities in this group are desmoplastic infantile astrocytoma (DIA) and ganglioglioma (DIG), papillary glioneuronal tumour, rosette forming glioneuronal tumour (RGNT), diffuse leptomeningeal glioneuronal tumour (also described as disseminated oligodendroglioma-like leptomeningeal neoplasm [42]), central neurocytoma and cerebellar liponeurocytoma. Apart from the anaplastic form of the ganglioglioma these lesions are well-differentiated, slow-growing neoplasms, which have distinct histologies, and varied genetic profiles. The *BRAF* V600E mutations occur in a proportion of gangliogliomas (see above) and rarely in DIA/DIG. Papillary glioneuronal tumours are rare, clinically benign and are characterised by a very specific chromosomal translocation resulting in a fusion oncogene *SLC44A1*–*PRKCA* [4]. RGNT occurs in the fourth and occasionally in the third ventricle and elsewhere periventricularly. They are rare, have a distinctive rosetting histological pattern and harbour mutations in the *PIK3CA* and *FGFR1* genes [17], but they currently have little diagnostic value as the histology is characteristic and unique in most instances. The rare diffuse leptomeningeal glioneuronal tumour has entered the 2016 update of the WHO classification as a new entity. Although it often shows histological similarities to oligodendroglioma, and harbours a solitary 1p or combined 1p/19q deletion, importantly it is not *IDH* mutant. *KIAA1549*–*BRAF* fusions can be present either alone or in conjunction with the 1p or 1p/19q deletions in diffuse leptomeningeal glioneuronal tumours [42]. An important differential diagnosis to this tumour is the pilocytic astrocytoma, which can have similar histological features (for example, clear cell morphology and leptomeningeal spread) and often harbours the *KIAA1549*–*BRAF* fusion. The central neurocytoma has a characteristic histology and apart from WNT pathway activation and *MYCN* amplification in some tumours, no characteristic mutations have been identified. Central neurocytoma can resemble, histologically, an oligodendroglioma, but can be molecularly easily discriminated as they have no *IDH* mutations or 1p/19q codeletion. A group of histologically relatively indistinct low-grade astrocytomas in children and young adults have emerged over the last years. These tumours do not harbour mutations in the *IDH*, *BRAF* or histone genes, but instead are characterised by *MYBL* gene rearrangements [37, 38, 60], which are thought to be driver mutations.

### Clinical relevance

Most of the low-grade glial and glioneuronal tumours are benign have distinct histologies and the molecular markers identified in research studies are therefore of limited diagnostic value. Given their rarity and relatively indolent clinical behaviour, it is also unlikely that these tumours will be on the priority list in the near future for the development of target-specific therapy against the disease causing genetic defect.

## Ependymomas

### Genetics and pathology

**Fig. 3:**
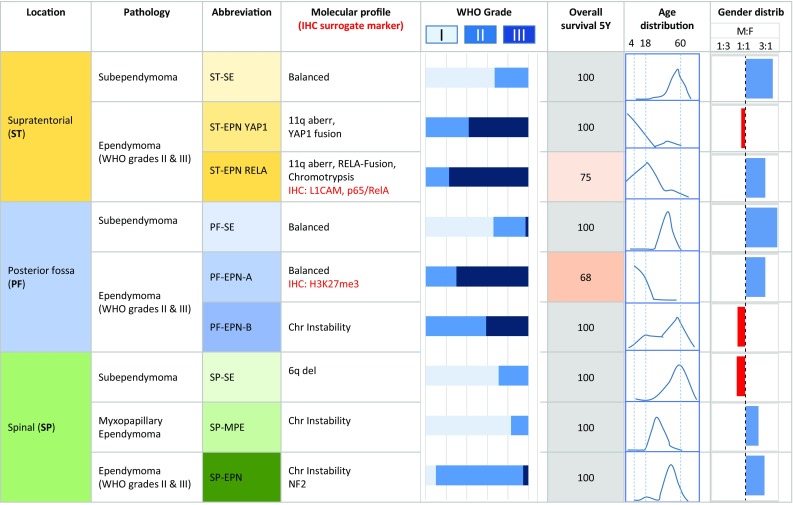
Diagrammatic summary of the most recent molecular subgrouping of ependymomas based on DNA methylation patterns [30, 31]. These molecular subgroups are genetically, epigenetically, transcriptionally, demographically and clinically distinct. Three topographically distinct overarching groups are identified, supratentorial (ST), posterior fossa (PF) and spinal (SP). Within each group there are three molecularly distinct molecular subgroups. Two of these nine groups are characterised by poor 5 year survival. The molecular profile column includes the immunohistochemically detectable surrogate markers of the PF-EPN-A and ST-EPN-RELA. All spinal tumours show favourable outcome when surgically completely removed

Ependymomas (and their variants subependymoma and myxopapillary ependymoma) are glial neoplasms predominantly arising within, or in the vicinity of, the ventricles and the spinal cord. The molecular classification of ependymomas has proven more meaningful for prognostication than the histological grading [31, 58], and although the nosological entity of anaplastic ependymoma is still recognised in the 2016 update of the WHO classification, it is clearly acknowledged that there is no association between histological grade and biological behaviour or survival. Over the last decade, distinct molecular subgroups of ependymomas have been demonstrated with various genetic techniques. Recently, a uniform molecular classification scheme has been proposed, based on DNA methylation profiling, resulting in nine distinct molecular subgroups (Fig. 3): three molecular groups are allocated to each, supratentorial, posterior fossa and spinal locations. In the supratentorial location, ependymomas with the presence of a fusion gene between *C11ORF95* and *RELA* (ST-EPN-RELA) have a poor prognosis, and in the posterior fossa location the ependymoma group PF-EPN-A is characterised by a poor prognosis. All other ependymomas [the two remaining supratentorial groups ST-EPN-YAP (YAP fusion) and ST-EPN-SE (subependymoma) and the two remaining infratentorial groups PF-EPN-B, PF-EPN-SE (subependymoma)] show a comparatively good prognosis [31]. As all spinal ependymomas have a good prognosis (when completely surgically resected), a molecular characterisation is not essential in routine clinical pathological practice. Surrogate markers have recently been identified for some of these subgroups and can be easily implemented into routine diagnostic practice. Immunohistochemical detection of the global reduction of H3K27me3 is highly sensitive and specific for PF-EPN-A and robustly discriminates them from PF-EPN-B, thus aiding prognostication [32]. The ST-EPN-RELA subgroup can be identified by immunostaining for the surrogate markers L1 cell adhesion molecule (L1CAM) or anti-NFkB (p65, RelA) [15], the latter based on the discovery that C11orf95–RELA fusions drive oncogenic NF-kB signalling in ependymoma [33].

### Clinical relevance

It is recommended that outside clinical trials, the WHO grading of ependymal tumours should not be used for the treatment decision [30]. Instead, the demonstration of nine clinically, demographically and molecularly distinct ependymoma entities offers new opportunities for (molecular) evidence-based treatments. A consensus publication [30] emphasises the controversy of the traditional histological grading system, and highlights the importance of the recognition of the molecular subgroups and mandates the use of the molecular classification of ependymomas for the enrolment in prospective clinical trials. However, as the recognition of the molecularly distinct subgroups is a recent development, no validated clinical trial data are available yet. This provides opportunities for the development of preclinical model systems and inclusion of advanced, genome wide molecular tests, such as methylation arrays and subsequent algorithmic classification into the routine diagnostics of supratentorial and infratentorial ependymomas.

## Conclusion and outlook

Many of the tumour entities described in this review are now defined by the presence of a mutation that serves as a biomarker, and there is now a consensus that certain brain tumour types should be diagnosed according to distinct biomarker profiles rather than histological features alone. It is striking, that the histological grade in some entities has become subordinate to the molecular profile, e.g. in ependymomas [31] and possibly also in IDH-mutant astrocytomas [40]. Continuous advances in next generation sequencing and methylation arrays will increasingly aid the diagnosis of some brain tumour entities [52] and help the prognostication of others [46]. A molecular classifying algorithm based on DNA methylation profile has been developed for most types of brain tumours, helping pathologists in establishing the diagnosis of histologically unusual tumours and to determine the molecular subclasses for example of medulloblastomas or ependymomas (http://www.molecularneuropathology.org). In those entities, e.g. Histone H3-, or IDH-wildtype glioblastomas, where no driver mutations are found, identification of molecular heterogeneity and characterisation of molecular subgroups in histologically similar tumours will help in the design of novel, more effective targeted therapies. The main clinical relevance of identifying specific (epi) genetic alterations in each tumour is in their potential to serve as target for inhibitor drugs, or for the development of therapies, such as immunotherapy with vaccines aiming at the destruction of cells expressing a mutant protein [48].
